# MRI features and preliminary diagnostic assessment using large language models of cystic tumor progression mimicking radiation necrosis in brain metastasis patients treated with immunotherapy: case report

**DOI:** 10.3389/fimmu.2025.1661918

**Published:** 2025-12-10

**Authors:** Guirong Tan, Mingchen Cai, Yu Lu, Amy Liu, Shimin Li, Gang Xiao, Lijuan Zhong, Lijia Li, Yichuan Hu, Qiong Liang, Haihui Jiang, Xiang Liu, Henry Z. Wang

**Affiliations:** 1 Advanced Neuroimaging Laboratory, Yuebei People’s Hospital Affiliated to Shantou University Medical College, Shaoguan, Guangdong, China; 2 Department of Radiology, Yuebei People’s Hospital Affiliated to Shantou University Medical College, Shaoguan, Guangdong, China; 3 Department of Medical Imaging, The Third Affiliated Hospital of Zhengzhou University, Zhengzhou, Henan, China; 4 Nazareth University, Rochester, NY, United States; 5 The Radiotherapy Center of Neoplasm, Yuebei People’s Hospital Affiliated to Shantou University Medical College, Shaoguan, Guangdong, China; 6 Department of Neurosurgery, Yuebei People’s Hospital Affiliated to Shantou University Medical College, Shaoguan, Guangdong, China; 7 Department of Pathology, Yuebei People’s Hospital Affiliated to Shantou University Medical College, Shaoguan, Guangdong, China; 8 Department of Pathology, The Third Affiliated Hospital of Sun Yat-Sen University, Guangzhou, Guangdong, China; 9 Department of Neurosurgery, Peking University Third Hospital, Beijing, China; 10 Department of Imaging Sciences, University of Rochester Medical Center, Rochester, NY, United States

**Keywords:** brain metastasis, tumor progression, radiation necrosis, immunotherapy, large language model, arterial spin labeling (ASL), dynamic susceptibility contrast perfusion-weighted imaging (DSC-PWI), immunotherapy response assessment in neuro-oncology (iRANO)

## Abstract

The application of immunotherapy in patients with brain metastasis (BM) has increased rapidly in recent years, which led to a diagnostic dilemma between pseudoprogression and true progression in the post-treatment evaluation. Here, we reported MRI characteristics of pathology-confirmed large cystic rim-enhancing lesions of tumor progression in two BM cases with immunotherapy treatment, which mimicked radiation necrosis on conventional MRI. In addition, our preliminary findings showed that the custom large language models (LLMs) using ChatGPT and DeepSeek yielded correct diagnoses in these cases, which may suggest the potential utility of LLMs for the decision-making in this field.

## 1 Introduction

Immunotherapy has revolutionized the treatment of multiple solid tumors in the past decade. The immunotherapy treatment used for tumor metastases in the brain from systemic cancers continues the rapid growth (1–4). The post-immunotherapy treatment imaging evaluation is very complex. It can be difficult to distinguish radiation necrosis and pseudoprogression from true progression given the similarity of conventional MR (5–10). Thus, a better understanding of imaging features, including a combination of advanced imaging techniques of MR perfusion-weighted imaging (PWI), of such brain metastasis (BM) patients with immunotherapy, will play an important role in the improvement of clinical management and prompt treatment decisions. In this article, we reported interesting MRI findings of pathology-confirmed atypical large cystic tumor progressions in two patients with BM following immunotherapy. This study was approved by the Ethics Committee of Yuebei People’s Hospital (approval No. KY-2022-042; approval date: May 30, 2022) and (approval No. YBEC-KY-2023-026; approval date: March 27, 2023). We also reported the preliminary diagnostic results using large language models (LLMs) (11).

## 2 MRI scan protocol and post-processing

MRI examinations were performed with a 1.5T (Signa HDxt) or 3.0 T MR scanner (Discovery 750, GE Healthcare, Milwaukee, WI, USA). Pre-contrast MRI protocol included axial T1- fluid-attenuated inversion-recovery (FLAIR), axial T2-weighted imaging, axial diffusion-weighted imaging (DWI), coronal T2-FLAIR, and three-dimensional pseudocontinuous arterial spin labeling (pCASL). DWI was performed using fat-suppressed single-shot spin-echo echo-planar imaging (TR/TE = 7,300/77 ms, slice thickness = 4.0 mm, slice gap = 0 mm, field of view = 220 × 220 mm, matrix = 130 × 160, NEX = 4) with b = 1,000 s/mm^2^ applied in the x, y, and z directions, and b = 0 s/mm^2^ without motion-probing gradients, and no distortion correction was applied. The pCASL was performed using a background suppressed 3D fast spin echo (FSE) technique. The parameters were as follows: TR/TE = 5,337 /10.7 ms; post-labeling delay (PLD) = 2.0 s; FOV = 240 × 240 mm; matrix = 512 × 512; slice thickness = 4.0 mm; slice gap = 0 mm; NEX = 3, labeling duration = 1.5 s.

The dynamic susceptibility contrast (DSC) PWI was performed with single-shot gradient-recalled echo-planar imaging (GRE-EPI) sequence (TR/TE = 1,525 ms/minimum, 250 Hz/pixel bandwidth, FOV = 240 × 240 mm, flip angle = 90°, matrix = 128 × 128, slice thickness = 4 mm, slice gap = 0 mm, NEX = 1). Fifty images were obtained for each slice. After ten phases, 0.1 mmol/kg Gd-DTPA (Guangzhou Consun Pharmaceutical Co., Ltd., Guangzhou, China) was injected at a rate of 5 ml/sec, immediately followed by a 20 ml bolus of saline at the same injection rate. Temporal resolution ≈ 1.5 s/volume. In the present study, a full-dose preload injection of contrast media before DSC-PWI acquisition was applied.

In addition, the susceptibility-weighted imaging (SWI) was performed in Case 1. Phase images were acquired using a three-dimensional flow-compensated gradient-echo sequence, slice thickness = 2.4 mm, TR/TE  =  42.5/22.7 ms, flip angle = 15°, FOV = 240 × 240 mm, matrix 384 × 288, NEX = 0.7.

Functional MRI data were post-processed using Functool software (AW 4.6, GE Healthcare, Milwaukee, WI, USA). The maps of apparent diffusion coefficient (ADC) and cerebral blood flow (CBF) were generated from DWI and ASL respectively. CBF quantified in mL/100 g/min using a single-PLD (PLD = 2.0 s) model assuming T1_blood = 1.6 s, T1_tissue = 1.2 s, labeling efficiency = 0.6 (a combination of both inversion efficiency 0.8 and background suppression efficiency 0.75 resulting in an overall efficiency of 0.6, λ = 0.9, with M0 obtained from reference image (PD) and corrected for partial saturation by using a T1_tissue of 1.2 s (typical of gray matter), processed on vendor-provided workstation (AW 4.6, GE Healthcare, Milwaukee, WI, USA). The cerebral blood volume (CBV) maps were generated using GE Brainstat arterial input function (AIF) model. The AIF was automatically identified in an arterial territory using the vendor provided built-in model, followed by quality control to ensure physiologically plausible curve shapes (rapid upslope, well-defined peak, and exponential washout). Voxels with abnormal time–intensity profiles or delayed bolus arrival were excluded. In the present study, the leakage correction algorithm was not applied.

**Figure 1:**
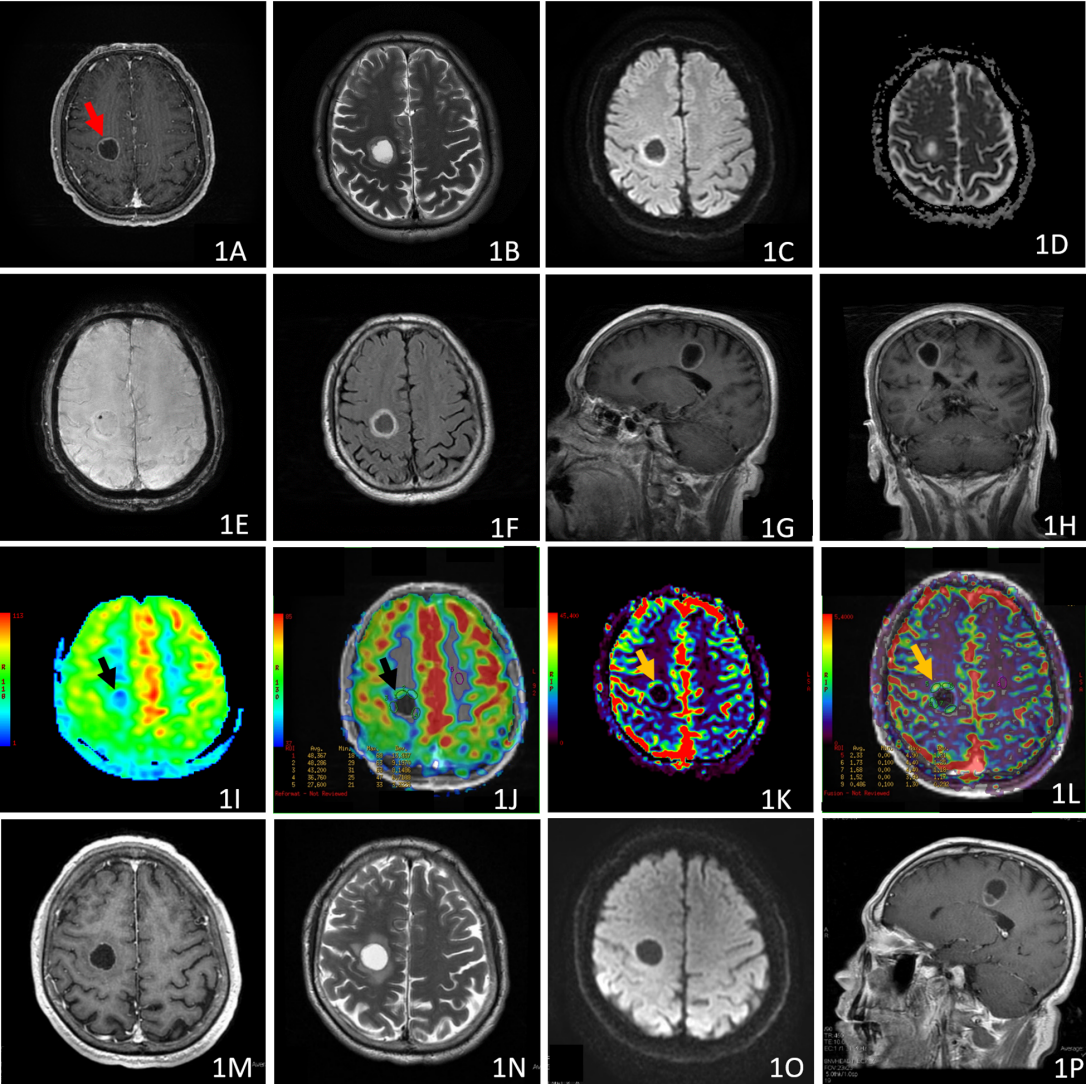
Case 1, axial post-contrast 3D T1-weighted **(A)**, T2WI **(B)**, DWI **(C)**, ADC **(D)**, SWI **(E)**, T2-FLAIR **(F)**, post-contrast sagittal T1-FLAIR **(G)**, and post-contrast coronal T1-FLAIR **(H)** of the pre-treatment MRI revealed a ring-enhancing mass without peritumoral edema in right frontal lobe. The enhancement of the rim was heterogeneous, with an enhancing nodule of the inner wall [red arrow, **(A)**]. The ASL-CBF maps [black arrow, **(I, J)**] and DSC-PWI-CBV maps [yellow arrow, **(K, L)**] showed increased perfusion of the anterior enhancing rim. Post-contrast 3D T1-weighted **(M)**, T2WI **(N)**, DWI **(O)**, and post-contrast sagittal T1-FLAIR **(P)**, of follow-up MRI which was scanned three months after immunotherapy and radiation treatment,showed decreased enhancement of the mass with slight edema. The mass volume did not change appreciably.

Semi-quantitative perfusion evaluation was obtained as described previously. First, four to six round or elliptic region of interest (ROIs), ranging in size from 50 to 85 mm^2^ each (>20 pixels), were placed in the enhancing tumor to record the maximal CBF/CBV value. An additional ROI was placed in the contralateral normal-appearing white matter as a reference (the **Supplementary Figure 1**; **Figure 1**). The maximal ratios of CBF and CBV (rCBF and rCBV) were calculated using the highest CBF and CBV values of enhancing tumor divided by CBF and CBV values of reference white matter, respectively (12). The placements of ROIs were conducted by two neuroradiologists with more than 10 years of experience in neuroradiology. Discrepancies were resolved by consensus with a third neuroradiologist (with more than 30 years of experience in neuroradiology). ROI placements were repeated on the same study after >2 weeks to assess repeatability, yielding inter-rater ICC = 0.913 for maximal rCBV and maximal rCBF.

## 3 Histopathological examination

The resection specimens were fixed in 10% neutral buffered formalin before being embedded in paraffin and sectioned at a thickness of 4 µm. The tissue sections were subjected to hematoxylin and eosin (H&E) staining as well as Immunohistochemical staining. Then, a diagnosis was made by an experienced pathologist based on the slides.

## 4 Case 1

A 60-year-old man was diagnosed with adenocarcinoma of the left lung, stage IV (pT4N3M1) after a biopsy. His pre-treatment brain MRI revealed a ring-enhancing mass in the right frontal lobe, (18.2 mm × 21.1 mm × 24.8 mm), without peritumoral edema, **Figure 1A–H**. The maximal thickness of the enhancing rim was 3.8mm.

Both the ASL-CBF and DSC-PWI-CBV maps showed elevated perfusion in the enhancing rim, with maximal rCBF of 1.83, and maximal rCBV of 4.67 (**Figures 1H–L**; **Supplementary Table 1**).

He was treated with gamma knife radiosurgery (margin dose of 18 Gy, 50% isodose, and maximal dose of 36 Gy) for his BM, and combination therapy of paclitaxel, cisplatin, and sintilimab (PD-1 inhibitor).

Three months later, his initial post-treatment MRI (**Figures 1M–P**) revealed a decrease in tumor enhancement. This mass presented mild peritumoral edema, and the tumor volume (18.5 mm × 19.4 mm × 23.4 mm) did not change dramatically compared to the pre-treatment MRI. The maximal thickness of the enhancing rim was 1.8 mm. These post-treatment MRI changes suggested a possible “Response” to the treatment of gamma knife radiosurgery and immunotherapy. Overall, his clinical examinations were stable, and he did not present headache and hemiparesis.

**Figure 2:**
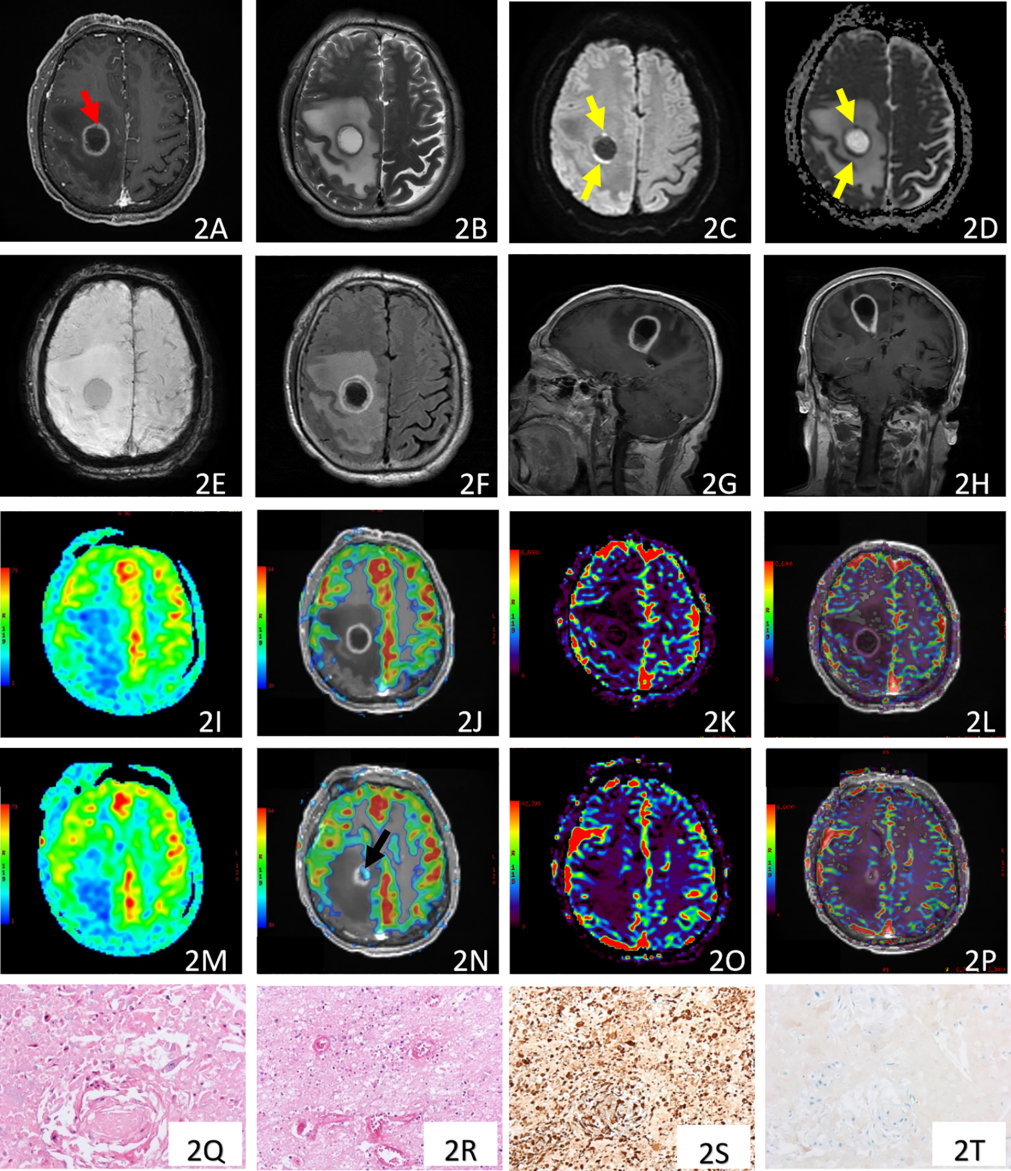
Case 1, a repeated MRI was performed two months later. Post-contrast 3D T1-weighted **(A)**, T2WI **(B)**, DWI **(C)**, ADC **(D)**, SWI **(E)**, T2-FLAIR **(F)**, post-contrast sagittal T1-FLAIR **(G)**, and post-contrast coronal T1-FLAIR **(H)** showed enlarged ring-enhancing mass and peritumoral edema. The enhanced rim showed patchy and linear restricted diffusion [yellow arrow, **(C, D)**]. The majority of this lesion did not present increased perfusion on both ASL-CBF and DSC-PWI-CBV maps (**(I–L, O, P)**. There was a nodule with increased ASL-CBF within the enhancement posterior to the right lateral ventricle [black arrow, **(M, N)**)], but without increased DSC-PWI-CBV **(O, P)**. Pathology images of case 1 with Hematoxylin-eosin (HE ×100) staining **(Q, R)**, and immunohistochemistry (×100) of CK5/6 **(S)** and GFAP **(T)**. **(Q)** showed heterogeneous cellular infiltration, necrosis, and keratinized beads. **(R)** showed hyperplasia of blood vessels and extravasation of erythrocytes. There were strongly positive expressions of CK5/6 in tumor cells in **(S)**. **(T)** revealed negative expression of GFAP in tumor cells.

Two months later, this patient presented headache and hemiparesis. His second post-treatment MRI was performed two months later (**Figures 2A–H**), which showed enlargement of this ring-enhancing mass (25.6 mm × 27.3 mm × 38.7 mm), and peritumoral edema. This large cystic lesion with a relatively smooth enhancing rim (the maximal thickness of the enhancing rim was 4.9 mm), accompanied by enlarged peritumoral edema, highly suggested the possibility of “radiation necrosis”. The ASL-CBF and DSC-PWI-CBV maps demonstrated decreased perfusion within the majority of the enhancing mass (maximal rCBF of 0.95, and maximal rCBV of 0.55, **Figures 2I–L, O, P**; **Supplementary Table 1**), which also supported the post-treatment imaging changes of radiation necrosis. However, ASL-CBF maps showed localized new enhancing nodules with increased CBF, with maximal rCBF of 2.02, **Figures 2M, N**, (maximal rCBV of the same region was 0.937, **Supplementary Table 1**). In addition, the enhanced rim showed patchy and linear restricted diffusion. These imaging features suggested potential tumor progression. His symptoms declined quickly, he progressed to hemiplegia, severe headache and nausea. This mass was resected nine days later. The pathology examination (**Figures 2Q–T**) showed extensive necrosis, within which there were active tumor cells consistent with metastatic squamous cell carcinoma.

## 5 Case 2

**Figure 3:**
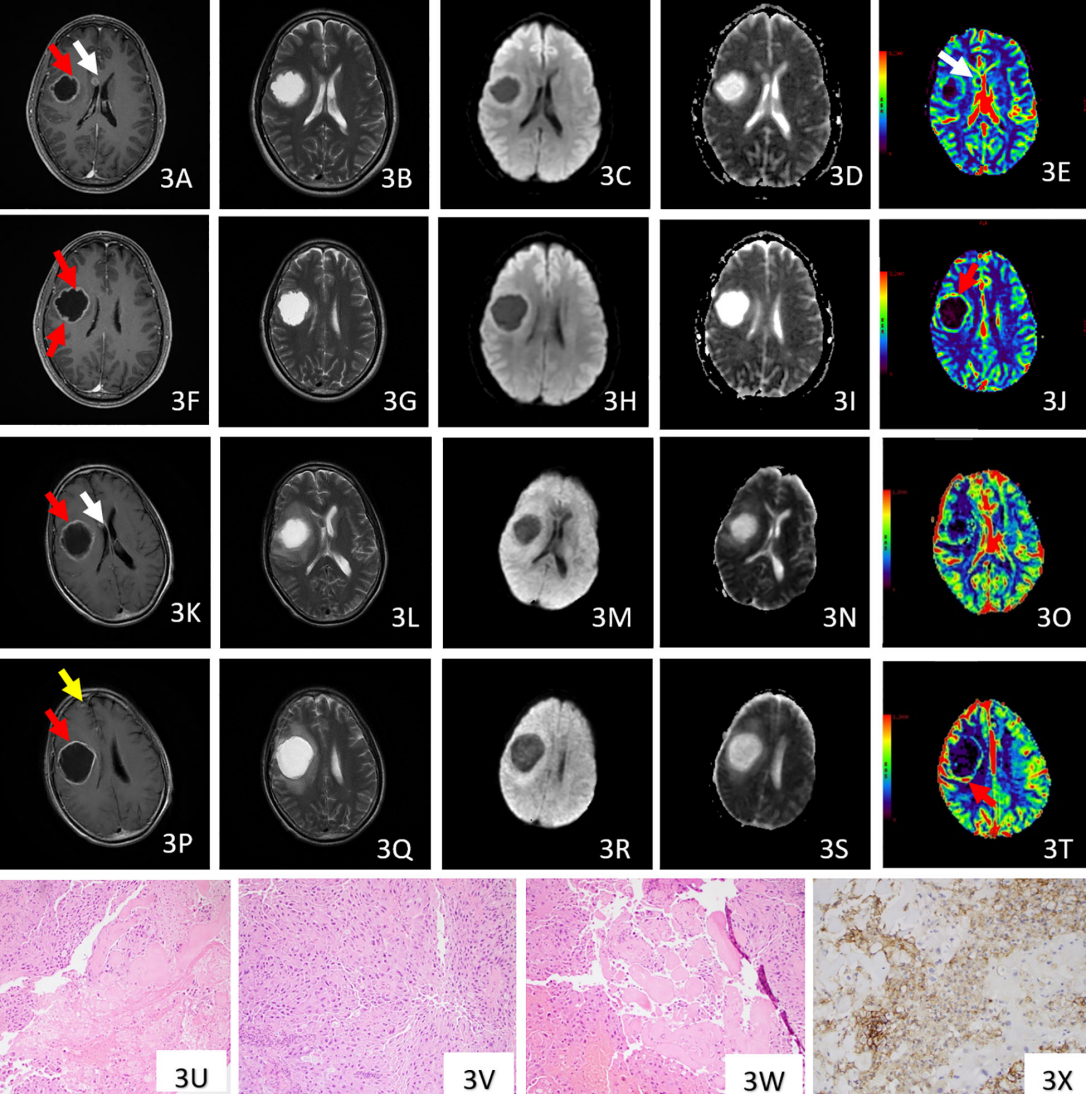
Case 2, post-contrast 3D T1-weighted **(A, F)**, T2WI **(B, G)**, DWI **(C, H)**, ADC **(D, I)** of pre-treatment MRI showed a right frontal ring-enhancing mass (red arrow) and a nodular enhancing tumor (white arrow) within the right lateral ventricle. The enhancement of the rim of the right frontal mass is heterogeneous, with multiple enhancing nodules. Both right frontal and right ventricle lesions presented increased perfusion on DSC-PWI-CBV maps **(E, J)**. **(K, T)** were MRI images of follow-up MRI two months after radiation and immunotherapy treatment. Post-contrast 3D T1-weighted **(K, P)**, T2WI **(L, Q)**, DWI **(M, R)**, ADC **(N, S)** showed that the right ventricle mass decreased (white arrow). The ring-enhancing tumor enlarged (red arrow) with increasing peritumoral edema. In addition, a new patchy enhancement (yellow arrow) was noticed in the right frontal lobe. On DSC-PWI-CBV maps **(O, T)**, the majority of the ring-enhancing tumors presented decreased perfusion compared to **(E, J)**. However, the posterior rim showed elevated CBV in **(T)**. Pathology images of case 2 with Hematoxylin-eosin (HE ×100) staining **(U–W)**, and immunohistochemistry (×100) of PD-L1 **(X)** showed extensive necrosis, heterogeneous cells (favor adenocarcinoma) and hyalinized vessels. **(X)** showed a high expression of PD-L1 of 60%.

A 58-year-old man was found with multiple BM (**Figures 3A–J**) after his lung adenocarcinoma was diagnosed by lung biopsy. The pre-treatment MRI revealed the largest brain mass was a ring-enhancing mass in the right frontal lobe, (37.8 mm × 42 mm × 34.3 mm), with mild peritumoral edema. The enhancement of the rim of this lesion was heterogeneous, and the maximal thickness of the enhancing rim was 10 mm. There was another nodular enhancing lesion along the right lateral ventricle. Both lesions presented elevated perfusion, with maximal rCBV of 2.14 and 1.6 respectively, **Supplementary Table 1**. This patient accepted whole-brain radiation treatment followed by Pembrolizumab. Two months later, the follow-up MRI, **Figures 3K–T** showed a decreased size of the right ventricle lesion. However, the right frontal ring-enhancing lesion enlarged (44.3 mm × 50.3 mm × 36.6 mm), with the maximal thickness of the enhancing rim decreased to 5.5 mm. The peritumoral edema also deteriorated compared to the pre-treatment MRI. These conventional MRI imaging findings favored the diagnosis of “radiation necrosis”. The DSC-PWI-CBV maps showed decreased CBV within the majority of the enhancing rim, consistent with post-treatment change. But the posterior rim showed elevated CBV with maximal rCBV of 2.79, **Supplementary Table 1**; **Figure 3T**, suggesting the “tumor progression”. This patient developed new seizure and left-sided facial weakness. A right-sided craniotomy was performed two weeks later. The pathology diagnosis (**Figures 3U–X**) was consistent with metastatic poorly differentiated adenocarcinoma.

## 6 Large language model analysis

Within the past three years, LLMs demonstrate significant potential in clinical medicine by enhancing decision support, diagnostics, and medical education (13–16). Previous studies showed the utility of LLMs in the radiology diagnosis assistance and interpretation of radiology reports (14, 17). Recently, Ozenbas et al. compared ChatGPT-4o with experienced radiologists in brain tumor MRI diagnosis, highlighting the diagnostic potential of LLMs in neuro-oncology (18). In the present study, we evaluated the diagnostic performance of LLMs including ChatGPT and DeepSeek.

The study was conducted between June 2 and June 4, 2025, and evaluated the diagnostic performance of four LLMs: ChatGPT-4o (OpenAI, released in May 2024), ChatGPT-o3 (OpenAI, released in April 2025), DeepSeek-V3 (DeepSeek, released in December 2024), and DeepSeek-R1 (DeepSeek, released in January 2025). All models were tested under their default configurations, with responses generated using the platform’s default decoding parameters (e.g., temperature and top-p).

**Figure 4:**
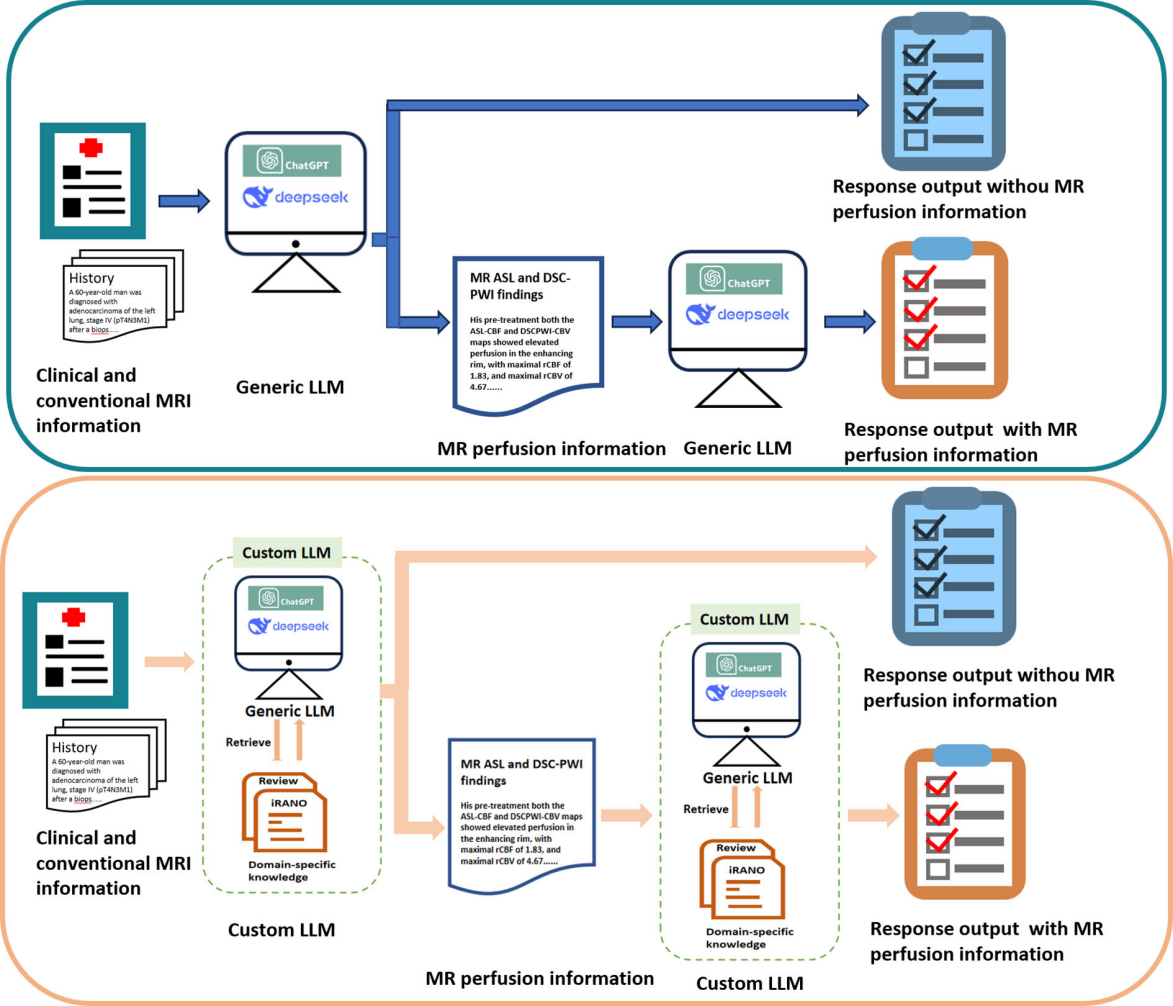
Flow diagram of LLM diagnostic workflow. Generic LLMs operate linearly: two case reports are provided to LLMs to generate responses. Custom LLMs integrate two case reports and domain-specific knowledge into the process. Generic LLM: ChatGPT-4o, ChatGPT-o3, DeepSeek-V3, and DeepSeek-R1; Custom LLM: ChatGPT-4o iRANO, ChatGPT-o3 iRANO, DeepSeek-V3 iRANO, DeepSeek-R1 iRANO; Domain-specific knowledge: the iRANO criteria and recent comprehensive neuroimaging review about brain tumors treated with immunotherapy.

Custom LLMs were built by combining generic LLMs with domain-specific knowledge and employing retrieval-augmented techniques. Firstly, two relevant literature including the immunotherapy response assessment in neuro-oncology (iRANO) guidelines (19) and recent comprehensive neuroimaging review about brain tumors treated with immunotherapy (20) were included as the domain-specific knowledge, and were input to the generic LLMs. By integrating the domain-specific knowledge with generic LLMs (ChatGPT-4o, ChatGPT-o3, DeepSeek-V3, and DeepSeek-R1), the custom LLMs (ChatGPT-4o iRANO, ChatGPT-o3 iRANO, DeepSeek-V3 iRANO, DeepSeek-R1 iRANO) were built, **Figure 4**. These custom LLMs retrieve and extract the most up-to-date and contextually relevant information from specific knowledge dataset, incorporate them with clinical and imaging information, provide more precise responses compared with generic LLMs.

Information from two case reports, including medical history, conventional MRI findings, and MR perfusion information (ASL and/or DSC-PWI), was de-identified. To ensure data confidentiality, all patient identifiers were removed, including names, dates, and institutional references. A neuroradiologist with 30 years of clinical experience edited all case materials and structured the input prompts to generate accurate queries (**Supplementary Table 2**).

The LLM analyses in case 1 and case 2 consisted of four tasks (**Figure 4**), and there were three diagnostic queries in each task, including Query 1: What is the diagnosis of the lesion? Query 2: Is this lesion tumor progression? And Query 3: Is this lesion pseudoprogression? In the first task, the generic LLMs were instructed to provide a diagnosis based on clinical information and conventional MRI findings, and each model was queried to determine whether the case represented true tumor progression or pseudoprogression. Second, MR perfusion imaging findings derived from ASL and/or DSC-PWI were added to the same case information, and the diagnostic queries were repeated to assess whether the additional perfusion information improved the diagnostic performance. Third, the custom LLMs were instructed to provide responses to the three queries using only clinical and conventional MRI findings. Finally, MR perfusion information was added to the input of the custom LLMs, and the diagnostic queries were repeated to evaluate the performance of the custom LLM integrated with tumor perfusion characteristics.

For consistency and reliability, each query was entered into a new chat session without prior context, and all model memory functions were disabled or cleared after each question to avoid recall bias. Two neuroradiologists independently evaluated the LLM-generated diagnostic interpretations. Inter-rater agreement was assessed using the ICC. Discrepancies were resolved by a third senior neuroradiologist through consensus discussion.

Representative responses generated from all LLMs can be found in **Supplementary Table 3**. Two neuroradiologists assessed the responses generated by LLMs and custom LLMs based on two criteria, 1) the answer should indicate the status of BM treated with immunotherapy, and 2) the final diagnosis should be “tumor progression” instead of “pseudoprogression”. Each task included three diagnostic queries, and each query was evaluated according to the above two criteria. Each query was scored on these two criteria, with 0 point= incorrect, 1 point= partially correct, and 2 points= correct and maximal score of every query is 2 points (**Supplementary Table 4**). As each task included three queries, the total score of each task ranged from 0 to 6 points. Finally, the diagnostic accuracy of all LLMs in each task was assessed using a five-point Likert scale (21), in which the total task scores (0–6) were converted as follows: scale 1 – completely incorrect (0 to 1 points), scale 2 – more incorrect than correct (2 points), scale 3 – approximately equal correct and incorrect (3 points), scale 4 – more correct than incorrect (4 points), scale 5 – correct (5 and 6 points), (**Supplementary Table 5**).

Two neuroradiologists independently scored all outputs; discrepancies were resolved by consensus with a third neuroradiologist. Inter-rater agreement was good (ICC = 0.883). In case 1, the custom LLMs of DeepSeek-R1 iRANO and ChatGPT-4o iRANO made “correct” diagnoses (received a score of 5 on the 5-point Likert scale). In case 2, the generic LLM of DeepSeek-V3, and the custom LLMs of DeepSeek-V3 iRANO and DeepSeek-R1 iRANO produced “correct” diagnoses (received a score of 5 on the 5-point Likert scale), **Supplementary Table 3**, **6**.

Descriptively, the custom LLMs (ChatGPT-4o iRANO and DeepSeek-R1 iRANO) produced higher median rubric scores than generic LLMs (ChatGPT-4o and DeepSeek-R1) across tasks in these two cases (**Supplementary Figure 2**, **Supplementary Table 7**).

The diagnostic performance scores were 3.50 (2.25, 5.00) when advanced MR perfusion imaging findings were provided, producing higher median scores than those without such findings [3.00 (3.00, 3.00)].

Further analysis in case 2 showed higher scores of all LLMs based on the information with advanced MR perfusion imaging findings than the scores based on the information without advanced MR perfusion imaging findings (5.00 (4.00, 5.00), 3.00 (3.00, 3.00), **Supplementary Table 8**.

## 7 Discussion

Cystic tumor is one of radiological features in brain tumors. The BM of lung adenocarcinoma can show cystic transformation (22). Essenmacher et al. reported one case with BM from adenocarcinoma of the lung developed multiple cystic brain lesions on surveillance MRI after erlotinib treatment. The radiological and pathologic characteristics of this case may relate to the effects of erlotinib on metastatic brain tumors (23).

Radiation necrosis and pseudoprogression are two adverse therapeutic effects after brain irradiation (7). The lung adenocarcinoma has been identified as one of the primary factors associated with radiation necrosis. The combination of immune checkpoint inhibitors with stereotactic radiosurgery has been reported to increase the risk of radiation necrosis (5–10). The morphological features of radiation necrosis and pseudoprogression, including the type of contrast enhancement, site, edema, and mass effect, are very similar to those of true tumor progression. Therefore, early detection and the correct interpretation of adverse treatment effects and tumor progression are crucial for prompt and optimal therapeutic decisions (11–16).

DWI and MR PWI are two advanced MRI techniques most commonly used in clinical practice to discriminate tumor progression from radiation necrosis and pseudoprogression of BM.

DWI and ADC measure the random motion of water molecules. ADC is usually low in tumor tissue due to high cellularity with pleiomorphic nuclei and a denser network of cytoplasmic. There are studies demonstrated that significantly lower ADC may suggest tumor progression compared to radiation necrosis and pseudoprogression. However, this distinction is not universal (5–10).

DSC-PWI and ASL are two common perfusion imaging techniques to evaluate hemodynamic changes of brain tumors (24, 25). There are multiple studies recommended rCBV cut-off points in distinguishing radiation necrosis from tumor progression and tumor recurrence in intra-axial brain tumors, which are summarized in **Supplementary Table 9**.

Barajas et al. investigated 30 intra-axial metastatic lesions in 27 patients treated with stereotactic radiosurgery (SRS), including 20 lesions diagnosed as recurrent BM and 10 lesions diagnosed as radiation necrosis. They found that the rCBV cut-off point of 1.52 enabled the detection of recurrence with a sensitivity of 91.30% and specificity of 72.73% (26). In addition, they reported that all 6 lesions with rCBV values below 1.35 were confirmed as radiation necrosis.

Mitsuya et al. analyzed 27 patients with BM undergoing SRS. They found that the rCBV of 7 recurrent BM lesions was significantly higher, ranging from 2.1 to 10, compared to the group of radiation necrosis (21 lesions), in which rCBV ranged from 0.39 to 2.57. The optimal rCBV cut-off value was determined to be 2.1 providing a sensitivity of 100% and a specificity of 95% (27).

Huang’s study consists of 33 metastatic lesions in 26 patients, in which rCBV cut-off point of 2 showing 56% sensitivity and 100% specificity to distinguish tumor progression from radiation injury (28).

In a cohort of 46 patients that underwent gamma knife radiotherapy for BM, Wang et al. observed that the rCBV of true tumor progression (median was 3.2 in 33 lesions) was higher than the radiation necrosis group (median rCBV was 1.0 in 25 lesions). They determined that the best cut-off rCBV value of 2.12 yielded a sensitivity of 90.9% and a specificity of 96% (29).

Morabito et al. measured rCBV values of 55 lesions in 8 patients with an intra-axial primary brain tumor (6 glioblastomas, 1 oligoastrocytoma, and 1 sarcoma) and 20 patients with intra-axial BM (lung carcinoma, breast carcinoma, larynx carcinoma, colon cancer, prostate carcinoma, neuroendocrine tumor, and melanoma). They set an optimal cut-off point for rCBV at 1.23 for differentiating radiation necrosis with tumor recurrence with 88% sensitivity and 75% specificity (30).

The reported rCBV cut-off points distinguishing radiation necrosis from tumor progression and tumor recurrence vary across studies, ranging from 1.23 to 2.12 (26–30). These studies included subjects with BM and malignant gliomas treated with radiation therapy with or without chemotherapy. The optimal rCBV cut-off point distinguishing radiation necrosis and pseudoprogression from tumor progression and tumor recurrence in primary and metastatic tumors following immunotherapy and radiation therapy is still under investigation, as immunotherapy can enhance inflammatory phenomena after radiotherapy in brain tumors (9).

The two BM cases in the present study were treated with immunotherapy and radiation therapy. Although the morphological imaging findings are difficult to discriminate between radiation necrosis and pseudoprogression from tumor progression and tumor recurrence, both cases showed new post-treatment regions with increased perfusion, maximal rCBF of 2.02 in case 1, and maximal rCBV of 2.79 in case 2 respectively, compared to pre-treatment MR perfusion imaging. The MR perfusion characteristics in both cases suggested the tumor progression which was confirmed by surgical pathology.

It is interesting that the post-treatment maximal rCBF was 2.02 in case 1, in contrast, the maximal rCBV was 0.937 in the same MRI examination (**Figures 2M–P**). This indicates a “mismatch” of perfusion evaluation between rCBF derived from ASL and rCBV derived from DSC-PWI. This phenomenon may support the underestimation of rCBV by the imaging technical limitations, including magnetic susceptibility artifacts due to petechial hemorrhage caused by irradiation (7), or tumors located near blood vessels, air, and bone (8). The size of the metastases can also limit the technique, especially in cystic metastases, where the thin wall may result in an undetectable rCBV (9). The diagnostic value of ASL in this field should be investigated in further studies. Other advanced imaging techniques, such as PET CT scan, are currently under investigation to distinguish radiation necrosis from tumor progression for BM treated with radiosurgery (31), and for assessing tumor response to immunotherapy (32–34).

It should be noted that biopsy with histopathologic evaluation remains the gold standard to differentiate radiation necrosis and pseudoprogression from tumor progression and tumor recurrence (5). It is common for the co-existence of radiation necrosis with tumor recurrence (7), and the percentage is variable and possibly depends on the time of evolution (9). The histopathologic complexity results in the imaging challenge.

RANO guidelines were recommended to evaluate brain tumor treatment response. RANO working group published a series of RANO guidelines, including the response criteria for high-grade gliomas (RANO-HGG, Standard RANO) in 2010 (35), low-grade gliomas (RANO-LGG) in 2011 (36), the Immunotherapy RANO Criteria (iRANO) (19), and the RANO Criteria for BM (RANO-BM) (37) in 2015, and the Modified RANO Criteria (mRANO) in 2017 (38). The latest updated RANO guidelines were RANO 2.0 which includes both high-grade and low-grade gliomas, were published in 2023 (39).

In the present study, we selected the guidelines of iRANO, and relevant literature for the domain-specific knowledge to develop custom LLMs. The custom LLMs of DeepSeek-R1 iRANO, ChatGPT-4o iRANO, and DeepSeek-V3 iRANO made correct diagnosis. Descriptively, custom LLMs (ChatGPT-4o iRANO and DeepSeek-R1 iRANO) produced higher median rubric scores than generic LLMs (ChatGPT-4o and DeepSeek-R1) across tasks in these two cases. These findings indicate that the custom LLMs show the potential to improve diagnostic performance in real-world clinical settings. The custom LLMs enhance retrieval process through the integration of current guidelines and literature which ensure that clinicians have access to the most current and relevant information of immunotherapy treatment response assessments for BM.

MR perfusion imaging demonstrated increased perfusion within the present two cases, which provided potential diagnostic clues for the atypical tumor progression mimicking large cystic radiation necrosis. It is very interesting that the MR perfusion information did not improve the diagnostic performance in the LLM analysis of two cases compared to conventional MRI findings alone. The comparison analysis result of the case 2 showed the trend of improved diagnostic performance based on MR perfusion information (**Supplementary Table 8**). We speculate that case 1 had multiple MRI examinations after immunotherapy treatment, which is compatible with iRANO assessment algorithm, thus, the LLMs can yield correct diagnoses without MR perfusion information, then the added MR perfusion information can’t improve the diagnostic performance in case 1. Compared to serial post-treatment MRI examinations of case 1, the case 2 only had one MRI examination two months after immunotherapy treatment due to the quick deterioration, and surgical resection was performed. The limited and early post-treatment MRI information may result in that LLMs did not recognize the tumor progression of case 2 based on iRANO assessment algorithm. The increased perfusion in case 2, as the biomarker of tumor progression, subsequently improved the correct diagnosis in custom LLM (DeepSeek-R1 iRANO and DeepSeek-V3 iRANO).

Our preliminary findings showed that the custom LLM of ChatGPT-4o iRANO, and DeepSeek-V3 iRANO yield correct diagnoses in case 1 and case 2 respectively, the DeepSeek-R1 iRANO made correct diagnoses in both case 1 and case 2. These results indicate that both ChatGPT and DeepSeek have similar decision-making performance, and they exhibit distinct strengths. DeepSeek models showed the potential promising diagnostic capability by the limited and atypical information on a single case, this needs to be validated in future large studies.

The advantage of customized LLMs is characterized by domain-specific training, in contrast, the retrieval-augmented generation (RAG) is a powerful technique providing more accurate, context-aware outputs by integrating updated corpus of knowledge, thus can avoid hallucinations (40). The discussion about which are superior between customized LLMs and RAG-based models remains unanswered. The selection of optimal approach for various knowledge-intensive tasks in clinical practice may depend on multiple factors, such as the type of medical information, and the specific clinical context. We can compare the performance of the customized LLMs and RAG-based models in our future studies.

## 8 Conclusions

In brief, we reported two BM cases treated with immunotherapy, presenting large cystic true tumor progression that mimics radiation necrosis on conventional MRI and were difficult to distinguish from true tumor progression. To our knowledge, published case reports that describe MRI characteristics, including MR PWI, of such atypical tumor progressions in BM cases treated with immunotherapy are limited. In these two cases, MR PWI provided additional functional information, which may be useful for the differential diagnoses. In addition, our preliminary study revealing that ChatGPT and DeepSeek exhibit distinct strengths, the custom LLMs using ChatGPT and DeepSeek prompts may assist interpretation. These observations warrant evaluation in larger cohorts.

## Data Availability

The original contributions presented in the study are included in the article/**Supplementary Material**. Further inquiries can be directed to the corresponding authors.

## Supplementary material

Supplementary Figure 1 Representative images of perfusion measurements using ROIs in Case 1. **(A, C)** are post-contrast 3D T1-weighted images, **(B)** is ASL-CBF map superimposed to axial post-contrast 3D T1-weighted image, and **(D)** is DSC-PWI-CBV map superimposed to axial post-contrast 3D T1-weighted image. On ASL-CBF map and DSC-PWI-CBV map, there were four green ROIs (approximately 73-80mm^2^) were placed within the enhancing tumor rim, and additional purple ROI was placed within contralateral white matter as reference.

Supplementary Figure 2 Bar charts of comparison analysis of mean scores between generic LLMs and custom LLMs.
