# Supplementary material for: MRI features and preliminary diagnostic assessment using large language models of cystic tumor progression mimicking radiation necrosis in brain metastasis patients treated with immunotherapy: case report
Source: Front Immunol. 2025 Dec 10;16:1661918. doi: 10.3389/fimmu.2025.1661918 (PMC12727969; doi:10.3389/fimmu.2025.1661918)
Supplement: Supplementary file 1 [file Table1.docx]

Supplementary Table 1: MR perfusion measurement results in Case 1 and Case 2.

| Type | Pre-treatment MR perfusion measurement | | Post-treatment MR perfusion measurement | | | |
| --- | --- | --- | --- | --- | --- | --- |
| Case 1 | | | | | | |
| Case 1-Location | Pre-treatment enhancing tumor rim | | Post-treatment enhancing tumor rim | | Post-treatment new enhancing tumor nodule | |
| Case 1-MR perfusion parameters | maximal ASL-rCBF | maximal DSCPWI-rCBV | maximal ASL-rCBF | maximal DSCPWI-rCBV | maximal ASL-rCBF | maximal DSCPWI-rCBV |
| Case 1 Result | 1.83 | 4.67 | 0.95 | 0.55 | 2.02↑ | 0.937 |
| Case 2 | | | | | | |
| Case 2-Location | Pre-treatment tumor 1 | Pre-treatment tumor 2 | Pre-treatment tumor 1 | | Pre-treatment tumor 2 | |
| Case 2-MR perfusion parameters | maximal DSCPWI-rCBV | maximal DSCPWI-rCBV | maximal DSCPWI-rCBV | | maximal DSCPWI-rCBV | |
| Case 2 Result | 2.1 | 1.6 | 2.79↑ | | The lesion was too small to be evaluated | |

↑indicates increased rCBF/rCBV compared to the pre-treatment values.
