# Supplementary material for: MRI features and preliminary diagnostic assessment using large language models of cystic tumor progression mimicking radiation necrosis in brain metastasis patients treated with immunotherapy: case report
Source: Front Immunol. 2025 Dec 10;16:1661918. doi: 10.3389/fimmu.2025.1661918 (PMC12727969; doi:10.3389/fimmu.2025.1661918)
Supplement: Supplementary file 2 [file Table2.docx]

**Supplementary Table 2：Detailed prompts information for LLM-based diagnostic tasks.**

| **Case 1** | **Detailed prompts** |
| --- | --- |
| Prompt 1 | Assuming you are a neuroradiologist，please walk me through the process from differential diagnosis to the most likely disease step by step, based on the patient's information and conventional MRI findings that I am about to present. |
| Prompt 2 | History  A 60-year-old man was diagnosed with adenocarcinoma of the left lung, stage IV (pT4N3M1) after a biopsy.  conventional MRI findings  His pre-treatment brain MRI revealed a ring-enhancing mass in the right frontal lobe, (1.82 cm×2.11 cm×2.48 cm), without peri-tumoral edema. The maximal thickness of the enhancing rim was 0.38cm. Three months later, his initial post-treatment MRI revealed a decrease in tumor enhancement. This mass presented mild peri-tumoral edema, and the tumor volume (1.85 cm×1.94 cm×2.34 cm) didn’t change dramatically compared to the pre-treatment MRI. The maximal thickness of the enhancing rim was 0.18 cm. His second post-treatment MRI was performed two months later, which showed significant enlargement of this ring-enhancing mass (2.56 cm×2.73 cm×3.87 cm), and peri-tumoral edema. This large cystic lesion with a relatively smooth enhancing rim (the maximal thickness of the enhancing rim was 0.49 cm), accompanied by enlarged peri-tumoral edema. In addition, the enhanced rim showed patchy and linear restricted diffusion. |
| Prompt 3 | Based on the diagnostic results, is it true tumor progression? |
| Prompt 4 | Based on the diagnostic results, is it pseudoprogression? |
| Prompt 5 | The patient also had MR perfusion imaging examinations, his MR perfusion imaging results are as following. Based on the provided conventional MRI, ASL, and DSC-PWI findings, what is the most likely disease and differential diagnoses? Please provide the reasoning. |
| Prompt 6 | MR ASL and DSC-PWI findings  His pre-treatment both the ASL-CBF and DSCPWI-CBV maps showed elevated perfusion in the enhancing rim, with maximal rCBF of 1.83, and maximal rCBV of 4.67. His second post-treatment MRI was performed two months later, the ASL-CBF and DSCPWI-CBV maps demonstrated decreased perfusion within the majority of the enhancing mass (maximal rCBF of 0.95, and maximal rCBV of 0.55), which also supported the post-treatment imaging changes of radiation necrosis. ASL-CBF maps showed localized new enhancing nodules with increased CBF, with maximal rCBF of 2.02 (maximal rCBV of the same region was 0.937). |
| Prompt 7 | Based on the current imaging findings, is it true tumor progression? |
| Prompt 8 | Based on the current imaging findings, is it pseudoprogression? |

| **Case 2** | **Detailed prompts** |
| --- | --- |
| Prompt 1 | Assuming you are a neuroradiologist，please walk me through the process from differential diagnosis to the most likely disease step by step, based on the patient's information and conventional MRI findings that I am about to present. |
| Prompt 2 | A 58-year-old man was found with multiple brain metastases after his lung adenocarcinoma was diagnosed by lung biopsy.  conventional MRI findings  The pre-treatment MRI revealed the largest brain mass was a ring-enhancing mass in the right frontal lobe, (3.78 cm×4.2 cm×3.43 cm), with mild peri-tumoral edema. The enhancement of the rim of this lesion was heterogeneous, and the maximal thickness of the enhancing rim was 1 cm. There was another nodular enhancing lesion along the right lateral ventricle. This patient accepted whole-brain radiation treatment followed by Pembrolizumab. Two months later, the follow-up MRI, showed a decreased size of the right ventricle lesion. However, the right frontal ring-enhancing lesion enlarged (4.43 cm×5.03 cm×3.66 cm), with the maximal thickness of the enhancing rim decreased to 0.55 cm. The peri-tumoral edema also deteriorated compared to the pre-treatment MRI. |
| Prompt 3 | Based on the diagnostic results, is it true tumor progression? |
| Prompt 4 | Based on the diagnostic results, is it pseudoprogression? |
| Prompt 5 | The patient also had MR perfusion imaging examinations, his MR perfusion imaging results are as following. Based on the provided conventional MRI and DSC-PWI findings, what is the most likely disease and differential diagnoses? Please provide the reasoning. |
| Prompt 6 | MR DSC-PWI findings  The pre-treatment both lesions presented elevated perfusion, with maximal rCBV of 2.14 and 1.6 respectively. Two months later, follow-up MRI, The DSCPWI-CBV maps showed decreased CBV within the majority of the enhancing rim, consisting of post-treatment change. But the posterior rim showed elevated CBV with maximal rCBV of 2.79. |
| Prompt 7 | Based on the current imaging findings, is it true tumor progression? |
| Prompt 8 | Based on the current imaging findings, is it pseudoprogression? |
