# Supplementary material for: MRI features and preliminary diagnostic assessment using large language models of cystic tumor progression mimicking radiation necrosis in brain metastasis patients treated with immunotherapy: case report
Source: Front Immunol. 2025 Dec 10;16:1661918. doi: 10.3389/fimmu.2025.1661918 (PMC12727969; doi:10.3389/fimmu.2025.1661918)
Supplement: Supplementary file 7 [file Table7.docx]

**Supplementary Table 7：Comparison of diagnostic performance between generic LLMs and custom LLMs.**

| **LLMs Model** | **Generic LLMs** | **Cust****om LLMs** |
| --- | --- | --- |
| ChatGPT 4o (median [IQR]) | 3.00 [3.00, 3.75] | 4.50 [3.25, 4.50] |
| ChatGPT o3 (median [QR]) | 3.00 [2.25, 4.50] | 3.00 [1.50, 4.50] |
| DeepSeek V3 (median [IQR]) | 3.00 [3.00, 3.00] | 4.00 [1.50, 5.00] |
| DeepSeek R1 (median [IQR]) | 2.00 [1.00, 4.50] | 4.00 [3.00, 5.00] |

LLM: large language model; IQR: interquartile range. Continuous variables consistent with a normal distribution were presented as mean ± standard deviation, otherwise the median and quartile are used. Continuous variables not conforming to the normal distribution were compared by the Mann-Whitney U test.
