# Supplementary material for: MRI features and preliminary diagnostic assessment using large language models of cystic tumor progression mimicking radiation necrosis in brain metastasis patients treated with immunotherapy: case report
Source: Front Immunol. 2025 Dec 10;16:1661918. doi: 10.3389/fimmu.2025.1661918 (PMC12727969; doi:10.3389/fimmu.2025.1661918)
Supplement: Supplementary file 8 [file Table8.docx]

**Supplementary Table 8: Comparison of diagnostic performance between LLMs without provided MR perfusion information and LLMs with provided MR perfusion information.**

| **Case** | **Without provided** **MR perfusion information** | **With provided MR perfusion information** |
| --- | --- | --- |
| All case (median [IQR]) | 3.00 [3.00, 3.00] | 3.50 [2.25, 5.00] |
| Case 1 (median [IQR]) | 3.00 [3.00, 4.50] | 2.50 [1.00, 3.00] |
| Case 2 (median [IQR]) | 3.00 [3.00, 3.00] | 5.00 [4.00, 5.00] |

LLM: large language model; IQR: interquartile range. Continuous variables consistent with a normal distribution were presented as mean ± standard deviation, otherwise the median and quartile are used. Continuous variables not conforming to the normal distribution were compared by the Mann-Whitney U test.
