# Supplementary material for: MRI features and preliminary diagnostic assessment using large language models of cystic tumor progression mimicking radiation necrosis in brain metastasis patients treated with immunotherapy: case report
Source: Front Immunol. 2025 Dec 10;16:1661918. doi: 10.3389/fimmu.2025.1661918 (PMC12727969; doi:10.3389/fimmu.2025.1661918)
Supplement: Supplementary file 9 [file Table9.docx]

**Supplementary Table 9:** **rCBV cut-off points distinguishing radiation necrosis from tumor progression/tumor recurrence in published studies.**

| **Literature** | **Brain tumor** | **Patients** | **Groups** | **rCBV** | **Sensitivity** | **Specificity** |
| --- | --- | --- | --- | --- | --- | --- |
| Barajas et al. [26] | Brain metastases | 27 | Radiation necrosis (10) vs Tumor recurrence (20) | 1.52 | 91.30% | 72.73% |
| Mitsuya et al. [27] | Brain metastases | 27 | Radiation necrosis (21) vs Tumor recurrence (7) | 2.1 | 100% | 95% |
| Huang et al. [28] | Brain metastases | 26 | Radiation injury (10) vs True tumor progression (23) | 2 | 56% | 100% |
| Wang et al. [29] | Brain metastases | 46 | Radiation necrosis (25) vs True tumor progression (33) | 2.12 | 90.9% | 96% |
| Morabito, et al. [30] | Primary and metastasic brain tumor | 28 | Radiation necrosis (32) vs Tumor recurrence (33) | 1.23 | 88% | 75% |

CBV: cerebral blood volume;
