# Supplementary figures and images for: MRI features and preliminary diagnostic assessment using large language models of cystic tumor progression mimicking radiation necrosis in brain metastasis patients treated with immunotherapy: case report
Source: Front Immunol. 2025 Dec 10;16:1661918. doi: 10.3389/fimmu.2025.1661918 (PMC12727969; doi:10.3389/fimmu.2025.1661918)

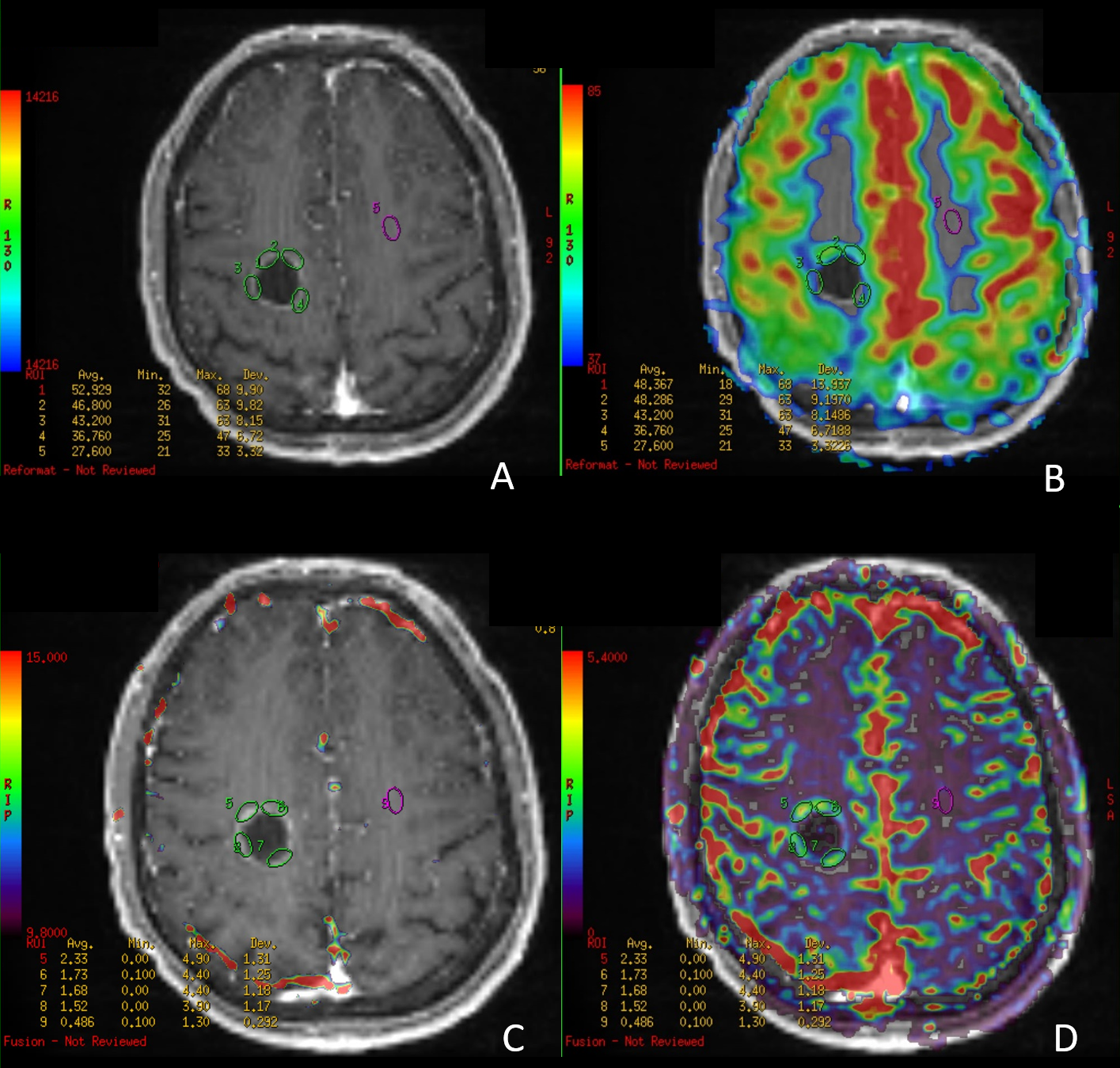

Supplement: Supplementary Figure 1 — Representative images of perfusion measurements using ROIs in Case 1. (A, C) are post-contrast 3D T1-weighted images, (B) is ASL-CBF map superimposed to axial post-contrast 3D T1-weighted image, and (D) is DSC-PWI-CBV map superimposed to axial post-contrast 3D T1-weighted image. On ASL-CBF map and DSC-PWI-CBV map, there were four green ROIs (approximately 73-80mm2) were placed within the enhancing tumor rim, and additional purple ROI was placed within contralateral white matter as reference. [file Image1.tif]

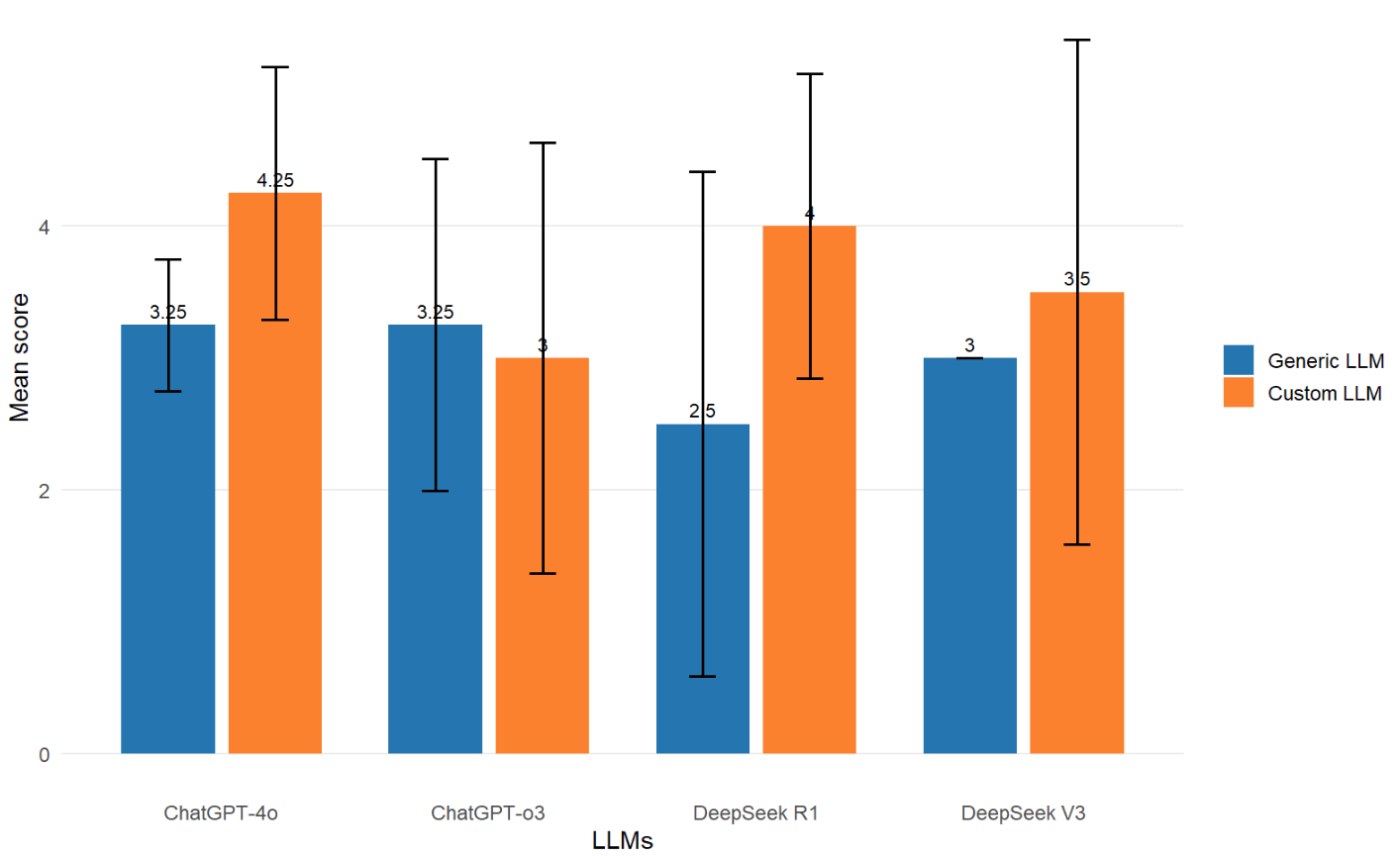

Supplement: Supplementary Figure 2 — Bar charts of comparison analysis of mean scores between generic LLMs and custom LLMs. [file Image2.tif]
